# Effect of workshops for coaches on the motor ability of balance in children practicing sports in late childhood

**DOI:** 10.1186/s13102-021-00388-9

**Published:** 2021-12-14

**Authors:** D. Wilczyńska, A. Łysak-Radomska, M. Podczarska-Głowacka, K. Krasowska, E. Perzanowska, A. Walentukiewicz, M. Lipowski, W. Skrobot

**Affiliations:** 1 Department of Physical Education and Social Sciences, Gdansk University of Physical Education and Sport, Kazimierza Górskiego 1, 80-336 Gdańsk, Poland; 2 Department of Health and Natural Sciences, Gdansk University of Physical Education and Sport, Kazimierza Górskiego 1, 80-336 Gdańsk, Poland

**Keywords:** Motor skills, Child athletes, Psychological influence

## Abstract

**Background:**

The coach attitude impacts the whole mind and body system of a child athlete from emotional and motivational aspects to motor skills like motor coordination. It translates into the ability to control and stabilize posture. The vestibular system plays an important role in this regulation. This system, next to the visual system and mechanoreceptors, is responsible for balance and control during posture transition. Moreover, the vestibular system is influenced by emotional factors. Therefore the authors of this study focused on the changes in the balance stability of children practicing sport after the implementation of the psychological workshops for coaches.

**Methods:**

Fifty-nine children at the age of 9–12 practicing soccer, art gymnastic and sport gymnastic were divided into two groups. The experimental group consisted of 31 participants and 28 in the control group. Experimental group children were under the influence of the 3 coaches who attended three workshops over 12 weeks period. Control group children were coached by 5 coaches who attended no workshops. Postural stability tests were performed on children before and after the workshops.

**Results:**

The statistically significant changes were observed in selected parameters of children’s balance stability after the experiment. The significant difference between the experimental and control group in Overall Stability Index (OSI) (*p* < 0.0002), Anterior–Posterior Stability Index (AP) (*p* < 0.01), Medial–Lateral Stability Index (ML) (*p* < 0.01) with eyes open were observed after the experiment. The results show a significant deterioration in control group contrary to the experimental group where the improvement trend was observed. Moreover, the difference between the groups was observed in OSI parameter (*p* < 0.005) with eyes closed after the experiment.

**Conclusion:**

The results obtained by the authors of the current study may support the thesis that educating coaches allows for better results in postural stability of child’s athletes. Nevertheless, this thesis requires further research, with particular emphasis on the age and gender of young athletes.

## Background

Coaches have a substantial effect on young athletes' physical and mental development [1]. The coaches' reactions and responses to children’s performance may affect the children's perception and evaluation of their competencies and general self-worth [2]. There are many types of interventions used to improve coaching style and mental support as well as to modify and increase the motivational climate to positively affect adult, child, and youth athletes’ well-being both in competition and in training [3–9]. In the present investigation, the intervention we used was workshops for coaches based on the i7W training model of Poczwardowski et al. [10]. This model intends to influence aspects of the psychosocial well-being of young athletes. The model is based on principles that form the Polish abbreviation i7W, representing an "i" and seven "w"s”: inspire (Inspiruj), explain (Wyjaśniaj), expect (Wymagaj), support (Wspieraj), reward (Wnagradzaj), and appreciate (Wyróżniaj), which all seem to have a positive effect on athletes and thereby contribute to growth (Wzrastać) and winning (Wygrywać). The results showed that athletes whose coaches practiced the i7W model in 8 workshops (lasting one and half hours each) estimated their relationships with coaches to be significantly stronger and their self-confidence and group cohesion to be higher. Athletes especially perceived an increase in their coaches' usage of behaviors from four categories: explain, reward, expect and appreciate [11]. Therefore, we decided to investigate the effects of such workshops with coaches of child athletes. While the coach's impact is highly substantial, we could also conclude that the coach's attitude impacts an athlete's whole mind and body system, from emotional and motivational aspects to essential motor skills such as motor coordination.

Motor coordination is an essential motor skill for maintaining physical and psychological health in childhood through adulthood [12]. Motor coordination consists of different abilities, and one of them is balance stability [13]. The control of body posture is defined as the ability to maintain the vertical position of the body in static or dynamic conditions [14]. The maintenance of balance depends on the proper functioning of the postural control system—the vestibular system, visual system, and mechanoreceptors from skin, muscles and joints [14–16]. Countless pieces of sensory information from the receptors of these systems continually reach the brain, where they are organized to be used for gravity safety, movement planning, and visual-spatial and auditory-linguistic processing as well as perception, postural maintenance, muscle tension, learning, emotional and social functioning and many others. The process of stimulus integration is a highly complicated process during which the recognition, segregation and interpretation of sensory stimuli occur as well as the comparison with previous experiences. The influence of vestibular stimulation on emotional states is mediated through vestibular system projections to the limbic system, hippocampus, insula, cingulate gyrus, and parabrachial nucleus via cerebellar and diencephalic centers, the brainstem, and amygdalar cells. Furthermore, the vestibular system is well networked with the dorsal raphe and the locus coeruleus, whose structures are valid for emotional state regulation [17]. Baccouch et al. [18], when investigating balance in young athletes practicing kung-fu and swimming, found that both sport disciplines develop balance skills that may be important for improving and staying in proper sport condition. The research shows that properly selected physical exercises as stimuli for the vestibular, neuromuscular and proprioceptive systems [19] may improve cognitive abilities, including memory, processing speed, executive functions, selective attention and spatial cognition. On the other hand, there is also evidence that mental training can contribute to the improvement of dynamic balance. The findings of Choi et al. [20] indicated that both physical balance training with visual feedback and mental balance training with motor imagery were effective for healthy adults. For instance, mental training based on visualization may influence the athlete’s functional state; moreover, when reducing or increasing psychophysical stimulation, visualization-based mental training may affect one’s emotional states [21]. Some studies suggest that mental training could be used effectively to improve motor skills or task performance because of its lower energy consumption than physical training [22]. Some authors underline that poor motor skills in children can be linked to low self-esteem and a fragile self-concept [23, 24]. Having an optimum level of self-esteem is crucial for self-confidence, goal accomplishment, the ability to act appropriately in difficult or important situations and stress coping [25]. Sabato et al. [26] emphasize that postural control and mental training to enhance self-esteem are injury prevention factors. Sampaio and Valentini [27] confirmed in their study that intervention programs for coaches and young gymnasts focused on climate mastery effectively promoted children's motor proficiency in fundamental motor skills and sport-related skills.

A body of studies indicates that coaches have a tremendous influence on many aspects of the mental characteristics of their athletes [28–31]; therefore, the authors of the current study found it reasonable to investigate the impact of psychological workshops for coaches on the postural control of their athletes.

## Method

### Participants

Participants of the study were children 9–12 years of age practicing soccer, art gymnastics and sport gymnastics. Participants were divided into two groups: an experimental group, including 31 participants, and a control group, including 28 participants. Children in the experimental group were under the influence of the 3 coaches who attended three workshops over a 12-week period. Children in the control group were coached by 5 coaches who attended no workshops. The characteristics of the groups are presented in Table 1.

**Table 1:** Characteristics of the participants

Characteristics	Experimental group	Control group
No. subjects	31	28
Age (years)	11.0 ± 1.19	11.53 ± 0.77
Height (cm)	145.39 ± 10.82	149.13 ± 6.51
Body weight (kg)	36.85 ± 9.99	41.10 ± 6.92
BMI	17.16 ± 2.83	18.37 ± 2.09
Skeletal muscle mass	16.63 ± 4.53	18.36 ± 2.97
Body fat mass	5.62 ± 4.7	6.86 ± 3.62

Values are mean ± SD

Moreover, we chose gymnastics and football because of their early specialization and the exclusion of other sports. Malina [32] and Waldron et al. [33] define early specialization as participation in a single sport discipline at or before the age of 12, with a high volume of training. In the current study, children from the control and experimental groups had similar experience in their sport (4.4 ± 1.6 and 4.5 ± 1.5 years, respectively) and similar experience with their current coach (3.1 ± 2.1 and 2.3 ± 1.6 years, respectively). The training sessions per week for the children in the experimental group and control group were also similar (5 ± 1.7 and 4.2 ± 0.9, respectively), and there were no differences in minutes of training per week between the experimental and control groups (119.2 ± 42.6 and 116.4 ± 41.6). All children were in the start of their competing period of the sport season.

### Design and procedures

The workshops were based on the i7W model of Poczwardowski et al. [10]. The activities in this model focus on the coach-player relationship to promote psychosocial development and sport achievement. As we mentioned before the model is based on principles that form the Polish abbreviation i7W, representing an "i" and seven "w"s”: inspire (Inspiruj), explain (Wyjaśniaj), expect (Wymagaj), support (Wspieraj), reward (Wnagradzaj), and appreciate (Wyróżniaj), which all seem to have a positive effect on athletes and thereby contribute to growth (Wzrastać) and winning (Wygrywać) [34]. The workshops aimed to increase the coaches’ ability to use the behavioral dimensions of the i7W model: inspire, support, explain, expect, reward, and appreciate.

There were three workshops, each lasting 6 h. The topics of the workshops are summarized in Table 2. For more information about the workshops, please contact the first author.

**Table 2:** Workshop topics (selected dimensions from i7W concept) and examples of the tools and activities introduced during the workshop

Workshop; topics	Tools and activities
First workshop; inspire, explain	“Pump them up”—prepare positive coach speech before your athletes' competition “Strengthening analysis”—conduct a post-match analysis with the athletes, focusing on the positive aspects of the game
Second workshop; expect, support	“Show your hand”—at the end of each week analyze simple goals that youth athletes formulated at the beginning of the week. Stimulate and help them to find answers to the following questions: How did I reach my goal? If not, what should I do differently in the future? “Positive bakes”—during training divide children into pairs. Each child from the pair is tasked throughout the training to provide verbal and non-verbal support to the teammate; e.g., giving high-five after a good performance, or a good word when teammate made a mistake. At the end of the training. ask the pair whether they felt the support of the teammate
Third workshop; reward, appreciate	“Good mistake”—Praise athletes despite them making mistakes. Show other youth athletes that creativity and courage is more important than making a single mistake. Highlight the fact that the biggest mistake one can make is not looking for the solution “Master T-shirt”– Introduce a special T-shirt for player who has shown the greatest commitment during training sessions

Table 2. Workshop topics (selected dimensions from the i7W concept), examples of the behaviors that coaches can exhibit and examples of the tools and activities introduced during the workshop.

Each workshop described a few dimensions to contribute to the “I grow and I win” concept of youth athletes. During each workshop, the coaches were taught practical applications and were provided with activities and a timetable to implement the i7W model. They were also provided with a template to record the activities they implemented. The workshops were organized in 6-h meetings over a 9-week period (one workshop every 3 weeks). Coaches sent the template to the coordinator of the study the day before the second and third workshops and 3 week after the last workshop.

**Fig. 1:**
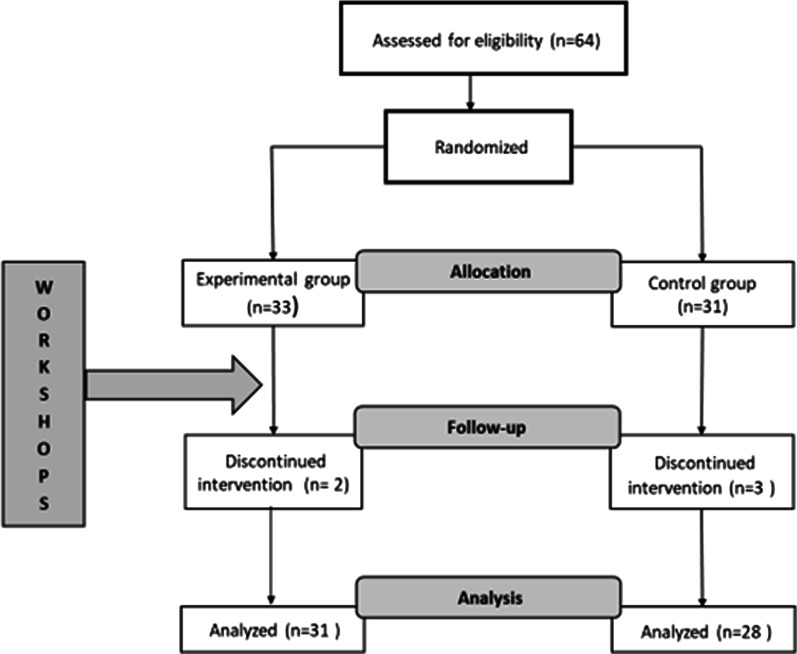
Flow diagram of the participants

Coaches were recruited by email contact with football and gymnastics clubs in the Pomeranian and Warmian-Masurian regions of Poland. Eight coaches from four clubs who expressed interest in participation were then sent an email with detailed information about the i7W workshop and the date of the first meeting. After the first meeting, two gymnastics coaches and six football coaches were randomly assigned to the control and experimental groups separately. (For the football coaches, randomization was stratified by club.) The flow diagram of the participants (young athletes) is described in Fig. 1. Eventually, 59 young athletes were examined after the last intervention (3 weeks after the last workshop for coaches).

The coaches in the experimental group were presented with more detailed information about the workshops, while those in the control group were told they would receive assessments of the balance characteristics of their athletes. After the coaches finally confirmed their participation in the study, they received information for the parents and guardians of the children about the aim, nature, and practice of the study.

The initial meeting with children took place in a research laboratory, where children were familiarized with all the tests required for the study. This familiarization took place one week before the first examination, and the authors—experimenters of the study described the balance stability examination, and children went through this test. The implementation of the study was then divided into three phases. In the first phase prior to the experiment (workshops), the experimental and control groups underwent balance tests in the laboratory. The second phase was the experiment itself. The workshops for coaches in the experimental group took place 3 times over 9 weeks period, when coaches practiced exercises during training with the participants prior to the second and third workshops and after the last workshop, the coaches gave feedback on practicing the i7W model (template). The control group of coaches attended no workshop at that time. The final phase took place in the laboratory, where the children underwent the same balance tests as prior to the intervention. Coaches were then sent anonymized reports based on a preliminary analysis of the balance performance of each child.

### Balance/postural stability examination

#### Biodex balance system (BBS)

The BBS is used to measure a subject’s ability to maintain balance. To assess postural stability the subjects underwent a postural stability test with the BBS (#950-440 System, Balance, SD 115VAC). The BBS software calculated a subject’s functional balance ability during a given task in terms of the anterior/posterior stability index, medial/lateral stability index, and overall stability index. These indices were calculated using the degree of oscillation of the platform, and low values showed that the subjects had good posture stability. The average of the three tests was considered the subject index.

The Postural Stability Test (PST) examines a subject’s ability to balance to maintain the center of gravity. In this test, the subject’s score depends on the number of deviations from the center; therefore, lower PST scores are better than higher scores. The subject had to keep the marker as close as possible to the center of the disk appearing on the screen. Each measurement was taken on a static platform at intervals of 20 s of testing and a 10-s break. The examined person stood on a static platform without shoes and with feet set freely apart. The age and height data of the subject were entered into the platform computer system. The subject's feet were placed on the platform, and the third metatarsal bone and the heel bone were considered. Taking the feet off and holding on were not allowed. The BBS enables an objective evaluation of postural stability through three indices: the overall stability index (OSI), anterior–posterior (AP) stability index and medial–lateral (ML) stability index. During tests, the platform was unstable.

Three tests were performed in a natural standing with bare feet. Two tests while standing on both legs and one single leg test. In the tests while standing on both legs, the measurement was performed with eyes open and eyes closed. Single leg test was performed with eyes open. During the first test, the platform stability index was 5; in the second test it was changed to a range of platform stability index from 8 to 1. A third test was performed on one leg (right and left) at platform stability index of 4 with eyes open.

### Statistical analysis

Statistica version 13.3 was used to analyze the data. Repeated-measures ANOVA was conducted to explore the effects of the intervention. Within-group comparisons between the pre- and posttests were performed by planned (a priori) contrasts for paired samples. The *p* value for the study was ≤ 0.05.

## Results

### Biodex balance system level 5

**Fig. 2:**
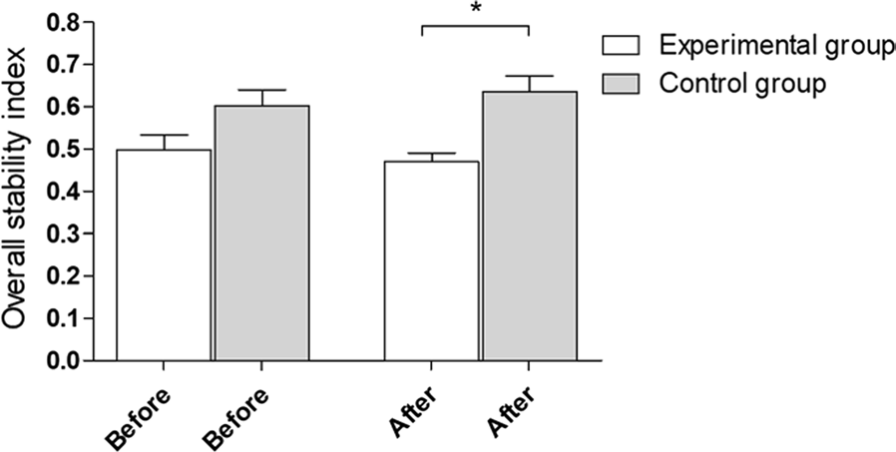
Overall stability index with eyes open—outcomes for 5 platform settings. Before—before experiment, after- after experiment

After the experiment, statistically significant (*p = 0.0002) between-group differences in the overall stability index with eyes open were observed. The mean and standard deviation before and after the experiment were 0.50 ± 0.19 and 0.47 ± 0.11 in the experimental group and 0.60 ± 0.2 and 0.64 ± 0.2 in the control group, respectively (Fig. 2).

**Fig. 3:**
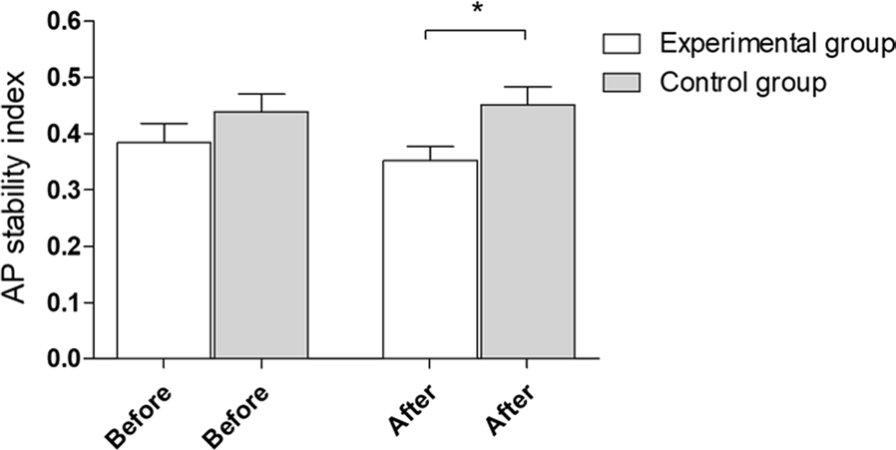
AP stability index with eyes open—outcomes for 5 platform settings. Before—before experiment, after—after experiment

After the experiment, statistically significant (*p = 0.01) between-group differences in the AP stability index with eyes open were observed. The mean and standard deviation before and after the experiment were 0.38 ± 0.19 and 0.35 ± 0.13 in the experimental group and 0.44 ± 0.16 and 0.45 ± 0.16 in the control group, respectively (Fig. 3).

**Fig. 4:**
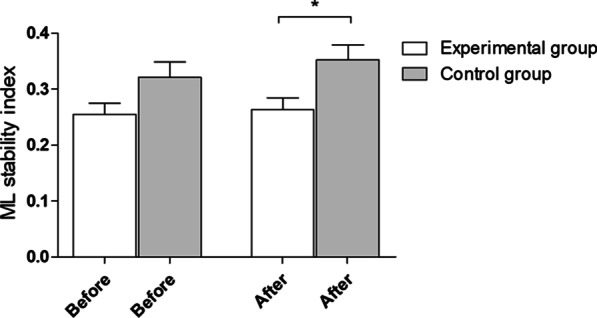
ML stability index with eyes open—outcomes for 5 platform settings. Before—before experiment, after—after experiment

After the experiment, statistically significant (*p = 0.01) between-group differences in the ML stability index with eyes open were observed. The mean and standard deviation before and after the experiment were 0.26 ± 0.1 and 0.26 ± 0.11 in the experimental group and 0.32 ± 0.14 and 0.35 ± 0.14 in the control group, respectively (Fig. 4).

After the experiment, statistically significant between-group differences in the overall, AP, and ML stability indices with eyes open were observed. In the control group after the experiment, a deterioration in the overall stability index with eyes open was observed. Simultaneously, after the experiment, statistically significant between-group differences were observed.

After the experiment for level 5 with eyes closed, no between-group differences in outcomes were observed.

### Biodex balance system level 8-1

**Fig. 5:**
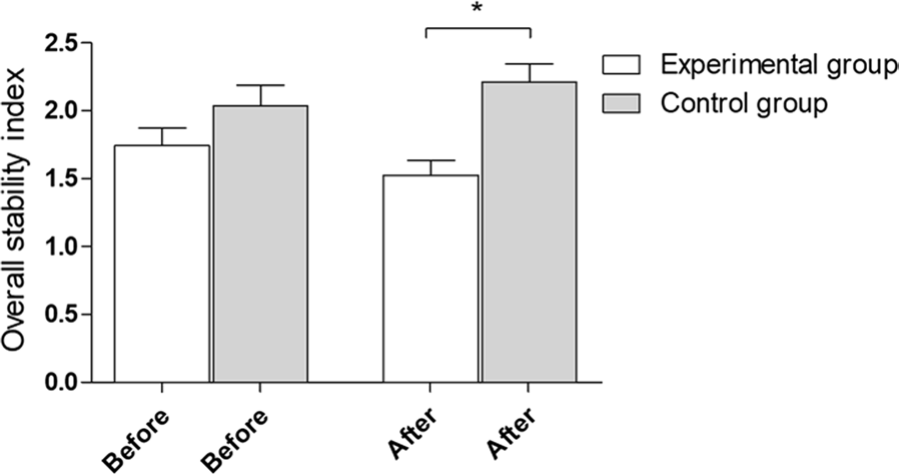
Overall stability index with eyes closed—outcomes for 8–1 of platform setting. Before—before experiment, after- after experiment

After the experiment, statistically significant (*p = 0.0005) between-group differences in the overall stability index with eyes closed were observed. The mean and standard deviation before and after the experiment were 1.74 ± 0.58 and 1.52 ± 0.48 in the experimental group and 2.04 ± 0.79 and 2.21 ± 0.71 in the control group, respectively (Fig. 5).

**Fig. 6:**
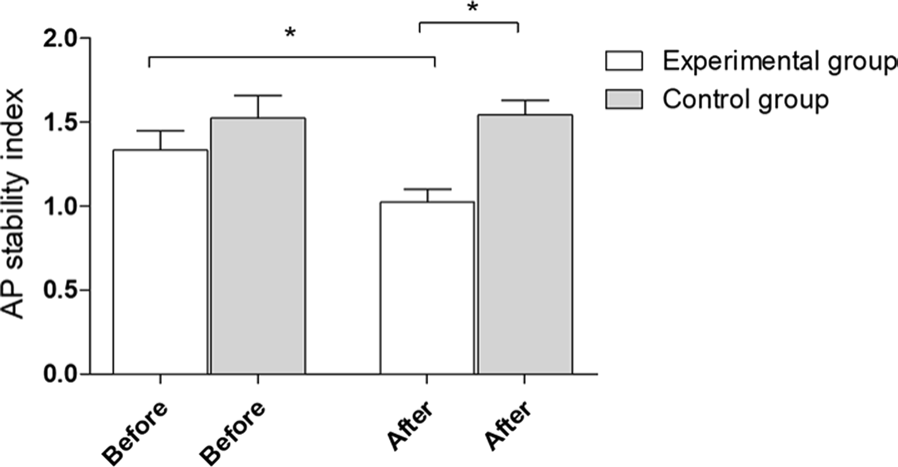
AP stability index with eyes closed—outcomes for 8–1 of platform setting. Before – before experiment, after- after experiment

After the experiment, statistically significant (*p = 0.0001) between-group differences in the AP stability index with eyes closed were observed. Simultaneously, in the experimental group, a statistically significant decrease in the AP stability index after the research protocol was observed (*p = 0.03). The mean and standard deviation before and after the experiment were 1.33 ± 0.52 and 1.02 ± 0.34 in the experimental group and 1.52 ± 0.79 and 1.54 ± 0.47 in the control group, respectively (Fig. 6).

**Fig. 7:**
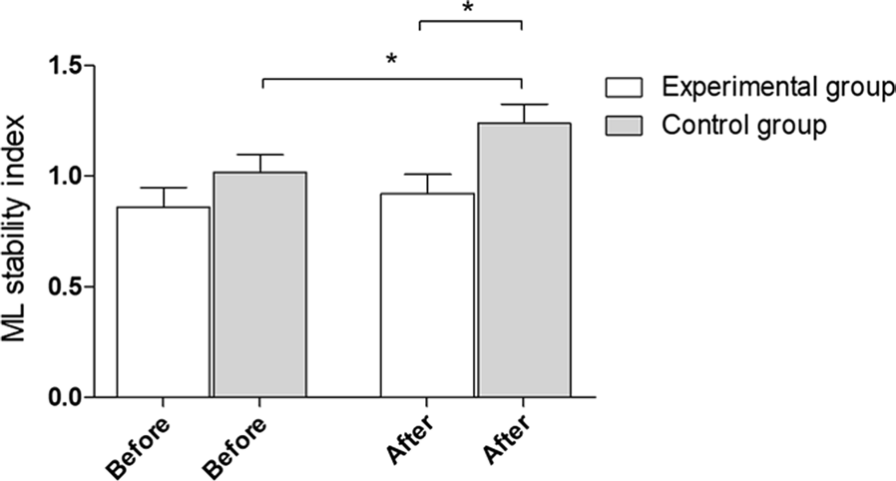
ML stability index with closed eyes—outcomes for 8–1 platform settings. Before—before experiment, after- after experiment

As mentioned above, after the experiment, statistically significant (*p = 0.01) between-group differences in the overall stability index with eyes closed were observed. Additionally, in the control group, a significant deterioration in the ML stability index was observed (*p = 0.01). The mean and standard deviation before and after the experiment were 0.86 ± 0.4 and 0.92 ± 0,38 in the experimental group and 2.04 ± 0,79 and 2.21 ± 0,71 in the control group, respectively (Fig. 7).

Overall, after the experiment, statistically significant between-group differences in the AP and ML stability indices with eyes closed was observed. Simultaneously, after the research protocol, a statistically significant decrease in the AP stability index was observed in the experimental group. Additionally, in the control group, a significant deterioration in the ML stability index was observed. After the experiment, no changes in outcomes with eyes open were observed.

## Discussion

Psychological workshops for coaches positively influenced selected parameters of stability control in children practicing sports. The authors noticed statistically significant changes in selected parameters of children’s balance stability after the experiment. There were significant differences between the experimental and control groups in the overall stability index (OSI), anterior–posterior (AP) stability index, and medial–lateral (ML) stability index with eyes open. The results also showed a significant deterioration in performance in the control group, contrary to the experimental group, where an improvement trend was observed. Moreover, a difference in the OSI parameter with eyes closed was observed after the implementation of the coaching workshops in the children in the experimental group.

Many factors can impair the quality of balance stability, such as the use of some medications; muscle weakness; pain; fatigue; depression; anxiety; and auditory, visual, and sensory stimuli and distractors [35]. If negative mental states can be detrimental to balance stability, we can expect that positive mental states will improve it. As long as there were no between-group differences in the training loads and ages of children in our study, we could assume that the i7W psychological workshops for coaches played a significant role in the stability progression of the experimental group children. The i7W model for coaches focuses on a holistic approach to an athlete’s career and especially on a positive approach to children and youth sports. The authors of the model underline that a coach who works with young athletes should plan a training session with thoughtful consideration of children’s and youth’s capacities and character using tools and drills from the *explain* and *support* factors of the i7W model. Also beneficial for the coach-athlete relationship are the activities from the *support* factor of the i7W model, which help the coach consider coping styles of young sport participants and adjust training tasks accordingly. When a coach is careful to ensure a positive coaching environment and conditions, children can thrive in sports and outside while maintaining joy and satisfaction [34]. The tools and drills used by coaches that influenced the motor balance of children in the current study probably came from selected factors of the i7W model such as *inspire*, *explain*, *support*, *reward*, and *appreciate*. Poczwardowski et al. [10] emphasize that the five factors mentioned above are the most crucial to develop in children and teenagers who are in the initiation and development phases of their athletic careers. Nevertheless, the study participants here were still in their late childhood; they were in the development phase because of their years of experience in practicing sports and sport competitions. In our opinion, the behaviors coaches learned during i7W workshops could impact motor ability, such as balance. As we mentioned above, parts of the workshops, especially when *inspire*, *explain*, *support*, *reward*, and *appreciate* concepts were presented, could impact children the most. During the first workshop, coaches were introduced to the *inspire* and *explain* concepts. The activities and tools recommended in the *inspire* part of the workshop help a coach to increase the positive vision of his young athletes and their development, to focus on personal, positive values to implement them in coaching methods, and to search for inspiration from other coaches who can successfully motivate their players. On the other hand, the *explain* part of the workshop teaches the coaches activities to support the individual abilities and strengths of their child athletes that can be used to perform complex tasks, to explain proper and expected behaviors, and to lead by example. This first part of the workshops could help develop young athletes' inner motivation and passion for sports, which follows. The ways the coach inspires athletes and explains things, which are aspects of coaching style, are crucial in the initiation phase of an athlete’s career, when children take their first steps in sports and competition [36]. In the second workshop, coaches were introduced to the *support* part of the i7W model. During this part, coaches learned activities that aim to strengthen the bond between them and their athletes, to place special care in the holistic development of the athletes and their individual development, and to increase the self-esteem and self-confidence of children through using positive feedback. We find examples in studies that low motor skills in youth athletes can be correlated with low self-esteem [23, 24]. The last workshop focused on the *reward* and *appreciate* concepts, when the coaches, apart from learning how to reinforce their child athletes, also concentrated on activities that improve team spirit, develop fair play behaviors in children, and emphasize the importance of team-supporting gestures in positive communication. This part of the workshop also focused on teaching coaches techniques to praise courage, creativity and persistence against all odds and mistakes. All activities and tools are mental techniques that could be used by a coach for the *growth* and *win* factors of the i7W model and factors that generally should be positively developed in sports and life. In summary, the mind and body are not inseparable, and a coach develops mental and physical aspects in their athletes the same dimension. Furthermore, the quality of this influence is remarkable and can have broad ramifications for a child's future career in sports [11, 34, 37, 38].

There are limitations of the study, and future research should address various aspects of design and implementation. First, the characteristics of the children's training and practice should be measured and described by the coaches, which can be accomplished by learning information on the training loads of the children and that they were in the starting period of their sport season. Additionally, workshops should evaluated to provide valuable information for the improvement of the workshops in the i7W model. Moreover, observing coaches during training sessions and sport competitions and meeting with them individually could reveal how well coaches implement the principles of the i7W model. A reasonable sample of coaches would permit an analysis that accounts for the nesting of children within coaches; coaching characteristics could then be assessed and included in the analysis to estimate coaching factors that modify the success of the workshops. Such factors might include personality, coaching style, and especially prior experience with the i7W model since it is reasonable to expect that some coaches in Poland have become familiar with the principles. If a sufficient sample of psychologists can be recruited, the psychologists who lead the workshops' personalities and experiences could also be investigated as modifying factors. Finally, future research should include follow-up in the postintervention period to determine the long-term effects of the workshops on child athletes' motor skills and other mental aspects.

## Conclusion

The results obtained by the authors of the current study may support the thesis that educating coaches in the i7W model allows better results of child athletes in terms of postural stability. Statistically significant changes were observed in selected parameters of children’s balance stability after the experiment. The significant deterioration in the control group, contrary to the experimental group, where an improvement trend was observed, allows the cautious conclusion that an appropriate positive approach toward the athlete can significantly influence some selected aspects of the motor skill of balance. Nevertheless, this thesis requires further research, emphasizing the age and sex of young athletes.

## Data Availability

All the data were presented in the manuscript. The raw data are available upon reasonable request to the corresponding author (DW).

## Abbreviations

AP: Anterior–Posterior Stability Index
BBS: Biodex balance system
i7W: Polish abbreviation representing an "i" and seven "w"s”: inspire (Inspiruj), explain (Wyjaśniaj), expect (Wymagaj), support (Wspieraj), reward (Wnagradzaj), appreciate (Wyróżniaj), growth (Wzrastać) and winning (Wygrywać)
ML: Medial–Lateral Stability Index
OSI: Overall Stability Index
PST: Postural stability test

## References

[1] Vella SA, Oades LG, Crowe TP (2013). The relationship between coach leadership, the coach–athlete relationship, team success, and the positive developmental experiences of adolescent soccer players. Phys Educ Sport Pedagogy.

[2] Horn TS (2019). Examining the impact of coaches’ feedback patterns on the psychosocial well-being of youth sport athletes. Kinesiol Rev.

[3] Barkoukis V, Tsorbatzoudis H, Grouios G (2008). Manipulation of motivational climate in physical education: effects of a seven-month intervention. Eur Phys Educ Rev.

[4] Boixadós M, Cruz J, Torregrosa M, Valiente L (2004). Relationships among motivational climate, satisfaction, perceived ability, and fair play attitudes in young soccer players. J Appl Sport Psychol.

[5] Bortoli L, Bertollo M, Filho E, di Fronso S, Robazza C (2017). Implementing the TARGET model in physical education: effects on perceived psychobiosocial and motivational states in girls. Front Psychol.

[6] Jaakkola T, Ntoumanis N, Liukkonen J (2016). Motivational climate, goal orientation, perceived sport ability, and enjoyment within Finnish junior ice hockey players. Scand J Med Sci Sports.

[7] MacDonald DJ, Côté J, Eys M, Deakin J (2011). The role of enjoyment and motivational climate in relation to the personal development of team sport athletes. Sport Psychol.

[8] Smith N, Quested E, Appleton PR, Duda JL (2017). Observing the coach-created motivational environment across training and competition in youth sport. J Sports Sci.

[9] Weigand DA, Burton SR (2002). Manipulating achievement motivation in physical education by manipulating the motivational climate. Eur J Sport Sci.

[10] Poczwardowski A, Serwotka E, Radomski K, Pogorselska A, Zienowicz A, Krukowska A (2015). Coaching in positive sport: theoretical bases of i7W model. Stud Sport Humanit.

[11] Krukowska A, Poczwardowski A, Kurach T et al. i7W model as a tool for coaches: theoretical and applied insights into the practice of positive sport. 2017.

[12] Opstoel K, Pion J, Elferink-Gemser M (2015). Anthropometric characteristics, physical fitness and motor coordination of 9 to 11 year old children participating in a wide range of sports. PLoS ONE.

[13] Raczek J, Mynarski W, Ljach W. Developing and diagnosing motor coordination abilities, 2nd edn. AWF Katowice; 2003.

[14] Pollock AS, Durward BR, Rowe PJ, Paul JP (2000). What is balance?. Clin Rehabil.

[15] Baghbani F, Woodhouse LJ, Gaeini AA (2016). Dynamic postural control in female athletes and nonathletes after a whole-body fatigue protocol. J Strength Cond Res.

[16] Yim-Chiplis PK, Talbot LA (2000). Defining and measuring balance in adults. Biol Res Nurs.

[17] Rajagopalan A, Jinu KV, Sailesh KS, Mishra S, Reddy UK, Mukkadan JK (2017). Understanding the links between vestibular and limbic systems regulating emotions. J Nat Sci Biol Med.

[18] Baccouch R, Rebai H, Sahli S (2015). Kung-fu versus swimming training and the effects on balance abilities in young adolescents. Phys Ther Sport.

[19] Angelaki DE, Cullen KE (2008). Vestibular system: the many facets of a multimodal sense. Annu Rev Neurosci.

[20] Choi JH, Choi Y, Nam KS, Cho IS, Hwang YT, Kwon YH (2010). Effect of mental training on the balance control ability of healthy subjects. J Phys Ther Sci.

[21] Wilczyńska D, Łysak A, Podczarska-Głowacka M (2015). Imagery use in rehabilitation after the knee joint arthroscopy. Balt J Health Phys Act..

[22] Lorey B, Bischoff M, Pilgramm S, Stark R, Munzert J, Zentgraf K (2009). The embodied nature of motor imagery: the influence of posture and perspective. Exp Brain Res.

[23] Coutinho MT, Santayana de Souza M, Valentini NC (2016). Crianças com desordem coordenativa desenvolvimental percebem-se menos competente e evidenciam autoconceito fragilizado/Children with Developmental Coordination Disorder perceived themselves less competent and showed a fragile self-concept. Rev Bras Ciênc E Mov RBCM..

[24] Yu JJ, Burnett AF, Sit CH (2018). Motor skill interventions in children with developmental coordination disorder: a aystematic review and meta-analysis. Arch Phys Med Rehabil.

[25] Suruba-Rusen AM, Murăreu DC (2019). Study on relationship between self-esteem and leisure activities in teenage sportsmen. Discobolul Phys Educ Sport Kinetotherapy J.

[26] Sabato TM, Walch TJ, Caine DJ (2016). The elite young athlete: strategies to ensure physical and emotional health. Open Access J Sports Med.

[27] Sampaio D, Valentini N (2015). Iniciação Esportiva Em Ginástica Rítmica: Abordagens Tradicional E O Clima Motivacional Para a Maestria [Youth rhythmic gymnastics: traditional approaches and motivational climate for mastery]. Rev Educ Física.

[28] Baker J, Côté J, Hawes R (2000). The relationship between coaching behaviours and sport anxiety in athletes. J Sci Med Sport.

[29] De Muynck GJ, Vansteenkiste M, Delrue J, Aelterman N, Haerens L, Soenens B (2017). The Effects of feedback valence and style on need satisfaction, self-talk, and perseverance among tennis players: an experimental study. J Sport Exerc Psychol.

[30] García-Naveira A, García-Mas A, Ruiz-Barquín R, Cantón E (2017). Intervention program based on coaching in young high performance athletes and their relationship with the perception of well-being and psychological health. J Sport Psychol.

[31] González L, García-Merita M, Castillo I, Balaguer I (2016). Young athletes’ perceptions of coach behaviors and their implications on their well- and ill-being over time. J Strength Cond Res.

[32] Malina RM (2010). Early sport specialization: roots, effectiveness, risks. Curr Sports Med Rep.

[33] Waldron S, DeFreese J, Pietrosimone B, Register-Mihalik J, Barczak N (2020). Exploring early sport specialization: associations with psychosocial outcomes. J Clin Sport Psychol.

[34] Poczwardowski A, Serwotka E, Radomski K, Pogorzelska A, Zienowicz A, Krukowska A (2015). Coaching in positive sport: theoretical bases of i7W model. Stud Sport Hum.

[35] Lord SR, Menz HB (2000). Visual contributions to postural stability in older adults. Gerontology.

[36] Ercegovac IR, Jukić T, Kegalj A (2020). The relationship between trainers’ coaching styles and young football players’ motivation. Res Kinesiol.

[37] Duda JL (2013). The conceptual and empirical foundations of Empowering Coaching^TM^: setting the stage for the PAPA project. Int J Sport Exerc Psychol.

[38] Duda JL, Quested E, Haug E (2013). Promoting adolescent health through an intervention aimed at improving the quality of their participation in Physical Activity (PAPA): background to the project and main trial protocol. Int J Sport Exerc Psychol.

